# Pulmonary perfusion with dynamic PEEP recruitment or sustained inflation at birth in preterm lambs

**DOI:** 10.1038/s41390-025-04183-x

**Published:** 2025-06-13

**Authors:** Joseph J. Smolich, Kelly R. Kenna, Magdy Sourial, Don Black, Anna Lavizzari, David G. Tingay

**Affiliations:** 1 Heart Research Group, Murdoch Children’s Research Institute, Parkville, VIC Australia; 2 Department of Paediatrics, University of Melbourne, Parkville, VIC Australia; 3 Translational Research Unit, Murdoch Children’s Research Institute, Parkville, VIC Australia; 4 Neonatal Research Group, Murdoch Children’s Research Institute, Parkville, VIC Australia; 5 Department of Critical Care, University of Melbourne, Parkville, VIC Australia; 6 NICU, Fondazione IRCCS Ca’ Granda Ospedale Maggiore Policlinico, Milan, Italy

## Abstract

**Background:**

Sustained inflation (SI) and dynamic PEEP recruitment (dynPEEP) aim to facilitate preterm lung aeration, but the effect of dynPEEP on pulmonary arterial (PA) blood flow after birth is unknown.

**Methods:**

Preterm (128 ± 1 day) fetal lambs instrumented with left PA and ductus arteriosus flow probes underwent positive-pressure ventilation (PEEP 8 cmH_2_O) after early cord clamping, preceded by either (1) SI at 40 cmH_2_O for 35 ± 3 s (*n* = 7) or (2) dynPEEP (*n* = 9) over 275 ± 23 s, comprising 2 cmH_2_O step rises in PEEP from 6 to 18 cmH_2_O followed by 2 cmH_2_O decrements to 6 cmH_2_O PEEP, then lung re-recruitment at 18 cmH_2_O PEEP. Hemodynamics were recorded for 30 min after birth.

**Results:**

During dynPEEP, PA blood flow increased linearly (*P* < 0.001) except for plateaus (1) between 12 cmH_2_O PEEP on the escalation limb and 14 cmH_2_O PEEP on the de-escalation limb, and (2) during lung re-recruitment. By contrast, PA flow increased during SI (*P* < 0.02), and was then briefly unchanged before rising linearly (*P* < 0.001). Consequently, post-birth rises in PA flow diverged between groups (*P* < 0.001), with this flow lower during dynPEEP by lung re-recruitment (*P* ≤ 0.048), but subsequently similar between groups.

**Conclusions:**

Only transient temporal differences in PA blood flow occur between SI and dynPEEP lung recruitment maneuvers at birth.

**Impact:**

- This study shows that a dynamic escalation and de-escalation positive end-expiratory pressure (PEEP) lung recruitment maneuver applied during the phase of rapidly-increasing pulmonary blood flow in the immediate period after preterm birth does not impede the peak of this flow increase, although transient plateauing of pulmonary flow occurs at high levels of PEEP
- This response contrasts with a sustained reduction of pulmonary blood flow reported during and after elevations in PEEP following stabilization of increased pulmonary perfusion after birth
- This preclinical study provides evidence that dynamic PEEP lung recruitment immediately after birth does not impair subsequent pulmonary perfusion

## Introduction

Initiation of effective tidal ventilation via rapid clearance of fetal lung liquid from the airways and into the lung interstitium, followed by aeration of alveolar saccules to increase lung volume and establish a functional residual capacity (FRC), is a fundamental feature of the birth transition.^1–3^ In structurally immature and surfactant-deficient preterm lungs, attainment of effective ventilation often requires positive pressure mechanical ventilation, with a role now well-established for positive end-expiratory pressure (PEEP) in augmenting FRC, avoiding alveolar collapse and preventing efflux of lung liquid back into alveolar spaces during expiration.^4–7^ FRC can be further improved in preterm lungs by incorporation of a brief sustained inflation (SI) prior to tidal ventilation, in order to overcome the high resistance and long time-constant arising from the presence of lung liquid within the airways.^4,8^ A dynamic PEEP (dynPEEP) recruitment maneuver, whereby PEEP is escalated and then de-escalated in a stepwise manner over several minutes immediately after the start of mechanical tidal ventilation, has also been shown to improve FRC and lung mechanics in preterm lambs.^9–11^

As well as promoting respiratory function, an SI may also augment pulmonary perfusion at birth, with a faster rise in mean pulmonary arterial (PA) blood flow reported in the initial minutes after the start of ventilation in preterm lambs.^12^ However, the effect of dynPEEP recruitment on the pattern of PA blood flow during the birth transition and whether the temporal features of the rise in this flow differ from those of an SI have not been addressed in any previous study. These issues are of particular relevance because large increases in PA blood flow at birth arise from the combination of (1) a redistribution of systolic right ventricular (RV) output towards the lungs, with a correspondingly lesser passage of this flow right-to-left (R→L) across the ductus arteriosus,^13,14^ and (2) left-to-right (L→R) and mainly diastolic shunting across the ductus, which emerges in the initial minutes after birth.^13–15^ Importantly, elevations in PEEP during mechanical ventilation of the already-aerated newborn lungs have been reported to (1) increase pulmonary vascular resistance and reduce both systolic and diastolic PA blood flow in preterm lambs,^16–18^ with reductions in PA blood flow persisting after cessation of the increased PEEP,^17,18^ (2) reduce RV output in ventilated preterm infants,^19,20^ and (3) increase R→L ductal shunting in newborn lambs^17^ and goats.^21^ Thus, the stepwise rises in PEEP which are an inherent component of the dynPEEP maneuver may potentially have adverse cardiovascular consequences after birth that include reductions in RV output and PA blood flow, and an increase in R→L ductal shunting.

This study, which was performed in anesthetized and acutely-instrumented preterm fetal lambs that were mechanically ventilated after cesarean section delivery and early clamping of the umbilical cord, therefore had two main aims. The first was to compare the effect of dynPEEP and SI maneuvers on the temporal pattern and magnitude of changes in PA blood flow, RV output and ductal shunting during the birth transition. The second was to specifically test the hypotheses that (1) elevations in PEEP during dynPEEP reduced PA blood flow and RV output, but increased R→L ductal shunting, and (2) PA blood flow changes persisted beyond the end of this maneuver.

## Methods

Studies conformed to guidelines of the National Health and Medical Council of Australia^22^ and were approved by the Murdoch Children’s Research Institute Animal Ethics Committee (Project A714). This manuscript is compliant with the ARRIVE guidelines for reporting of animal research (Checklist provided in Supplement 1).^23,24^

### Surgical preparation

The general features of the anesthetic and monitoring procedures were as previously described,^25,26^ with 16 Border-Leicester cross pregnant ewes anesthetized and monitored at a gestation of 128 ± 1 days (mean ± SD, term = 147 days) as detailed in Supplement 2.

The uterus was exposed via a midline laparotomy. With a twin pregnancy, the position of both fetuses was assessed by palpation, and the presence of any meconium staining in each fetus determined via a small uterine incision, usually over a hindlimb. The most accessible fetus in the best condition was then chosen for surgical preparation, with the other fetus completely delivered from the uterus and humanely killed with an intracardiac injection of sodium pentobarbitone (100 mg/kg) after clamping and cutting of the umbilical cord.

The head of the experimental fetus was exteriorized and placed in a saline-filled glove to prevent loss of lung liquid. Via a neck incision, catheters were inserted into the left external jugular vein for fluid and drug administration, and a 6-Fr vascular sheath was passed via the left common carotid artery into the brachiocephalic trunk for pressure measurement and sampling of ascending aortic/aortic trunk (AoT) blood. After exteriorization of the left forelimb and upper thorax, a thoracotomy was performed in the 3^rd^ left interspace. Following careful dissection, non-constrictive transit-time flow probes (Transonic Systems, Ithaca, NY) were placed around the left PA (size 4 or 6 mm) and ductus arteriosus (size 8 or 10 mm). To measure pressures, a fluid-filled catheter and a high-fidelity 3.5-Fr micromanometer catheter (SPR-524, Millar Instruments, Houston, TX) were inserted via purse-string sutures into the pulmonary trunk (PT) close to its junction with the ductus and common PA, and a fluid-filled catheter was introduced into the left atrial (LA) appendage. Following completion of surgery, the trachea was intubated with a clamped 4.5 mm cuffed endotracheal tube having a side-port at its connector end to measure tracheal pressure. Due to the presence of multiple catheters and flow probes, thoracic structures were positioned in their normal anatomical location at the end of surgery, but the thoracotomy was not surgically closed.

### Experimental protocol

The endotracheal tube was unclamped to allow lung liquid to drain passively via gravity for ∼30 s, simulating a reduction in lung liquid volume that normally occurs during the birth process.^27–29^ After re-clamping of the endotracheal tube, an AoT sample was withdrawn for blood gas analysis (ABL800, Radiometer, Copenhagen, Denmark), and computer recording of physiological data commenced. Lambs were then completely delivered from the uterus and the umbilical cord immediately clamped and cut. After weighing, lambs were transferred into a supine position on a heated neonatal resuscitation bed prior to either a dynPEEP or SI maneuver, with this start of this maneuver defined as the point of birth. The interval between cord clamping and the start of dynPEEP or SI did not differ significantly (41 ± 11 vs. 35 ± 7 s, *P* = 0.25, unpaired Student’s *t* test), with these brief intervals avoiding confounding hemodynamic effects arising from a rapid decrease in arterial oxygenation that reaches an asphyxial level if the cord clamp-to-ventilation interval exceeds ∼1 min.^13,30–32^ Allocation of lambs to the dynPEEP or SI groups was performed before the start of surgical preparation, with allocation mainly dependent on the availability of clinical or research staff required to perform these maneuvers.

In lambs undergoing dynPEEP (*n* = 9, 5 male and 4 female, 6 singletons and 3 twins) mechanical ventilation was commenced using a warmed and humidified O_2_/air mixture in a volume-targeted mode, a PEEP of 6 cmH_2_O, a maximum permissible peak inspiratory pressure (PIP) of 50 cmH_2_O, an inspiratory time (*T*_I_) of 0.4 s, an inspired O_2_ concentration (FiO_2_) of 0.3, a tidal volume (V_T_) of 7 ml/kg body weight, and a respiratory rate of 60 breaths/min. The lung was then recruited by increasing PEEP in steps of 2 cmH_2_O every 15–20 inflations up to a PEEP of 18 cmH_2_O, a level where peak dynamic lung compliance (C_dyn_) is attained in lambs at the gestation used in the present study.^33^ PEEP was then reduced by 2 cmH_2_O every 15–20 inflations until a PEEP of 6 cmH_2_O. Subsequently, PEEP was increased to 18 cmH_2_O for 15–20 inflations to transiently re-recruit the lung and then reduced to 8 cmH_2_O, with ventilation subsequently continued at ventilator settings described below. The duration of the dynPEEP maneuver (i.e. from the start of the initial PEEP of 6 cmH_2_O to the start of the final PEEP of 8 cmH_2_O) was 275 ± 23 s; no animal developed a pneumothorax during this maneuver.

In SI lambs (*n* = 7, 3 male and 4 female, 3 singletons and 4 twins), lungs were inflated via the endotracheal tube to a pressure of 40 cmH_2_O for 35 ± 3 s with an FiO_2_ of 0.3 using a Neopuff Infant T-piece Resuscitator (Fisher & Paykel Healthcare, Auckland, New Zealand) having a total flow of 8 l/min.^11,34^ The endotracheal tube was then transiently clamped to prevent lung volume loss and connected to an infant ventilator (SLE5000, SLE Ltd, Croydon, UK), with mechanical ventilation commenced at the ventilator settings described below.

Positive-pressure ventilation in all lambs was volume-targeted with ventilator settings comprising a PEEP of 8 cmH_2_O, a maximum PIP of 50 cmH_2_O, a respiratory rate of 60 breaths/min, a T_I_ of 0.4 s, a V_T_ of 7 ml/kg body weight and an FiO_2_ of 0.3. These settings are physiologically appropriate for the gestational age of lambs used in the present study.^9^ Ventilator settings were subsequently adjusted as required to attain a preductal oxygen saturation, measured with a pulse-oximetry sensor on a cheek, of >90% by 10 min after birth.^35^ After delivery, anesthesia in lambs was continued with an i.v. infusion of ketamine (4–8 mg/kg/hr) and midazolam (0.05–0.1 mg/kg/hr).

The recording of physiological data that was commenced just prior to delivery of the lamb was stopped ∼10 min after birth, preceded by withdrawal of an AoT sample for blood gas analysis. Hemodynamic and blood flow data were subsequently collected at 15 and 30 min after birth, with each recording accompanied by withdrawal of an AoT blood gas sample. At the 10 min time-point and beyond, ventilator settings were adjusted on the basis of AoT blood gas results, with a target hemoglobin O_2_ saturation (S_a_O_2_) of >90% and CO_2_ tension (P_a_co_2_) of 40–50 mmHg, to mimic the clinical situation where ventilation is adjusted on the basis of individual patient needs.

A priori criteria for inclusion of experimental studies were that full fetal instrumentation with pressure catheters and flow probes was able to be performed, and that instrumented animals underwent an uneventful dynPEEP or SI maneuver after an uncomplicated cesarean section birth delivery. Animals were humanely killed with an i.v. overdose of sodium pentobarbitone (100 mg/kg), administered to ewes shortly after cord clamping, and to lambs after completion of the study protocol. At postmortem, no differences were present between dynPEEP and SI lambs in body weight (3.81 ± 0.48 vs. 3.63 ± 0.45 kg, *P* = 0.455, unpaired Student’s *t* test), total lung weight (126 ± 23 vs. 118 ± 24 g, *P* = 0.495) or the total lung-to-body weight ratio (33.1 ± 4.4 vs. 33.0 ± 8.4 g/kg, *P* = 0.980), but the total-to-left lung weight ratio tended to be lower in dynPEEP lambs (2.39 ± 0.21 vs. 2.56 ± 0.10, *P* = 0.054).

### Physiological data

AoT, PT, LA and tracheal catheter pressures were measured with transducers referenced to atmospheric pressure at LA level. Catheter, micromanometer and flow probe signals were digitized at a sampling rate of 1 kHz using programmable acquisition and analysis software (Spike2, Cambridge Electronic Design, Cambridge, UK). All experimental studies had blood flow signals of sufficient quality for accurate perinatal determination of the primary physiological measures of interest (PA flow, net ductal flow, phasic L→R and R→L ductal flows, RV output).

Fio_2_, V_T_, PIP and C_dyn_ were recorded at 10, 15, and 30 min after birth from the ventilator display, and the alveolar-arterial O_2_ difference (A-aDO_2_) calculated using a standard formula.^6^

Following onset of mechanical ventilation in the dynPEEP group, data epochs 5–7 s in duration were extracted from the main birth data file at each of the 15 sequential levels of PEEP in this maneuver. Data epochs of similar duration were obtained at corresponding mean time-points in SI lambs, starting from the onset of SI. Note that, with this analysis protocol, the timing of the SI corresponded to the initial 6 and 8 cmH_2_O PEEP steps in dynPEEP. In both groups, 10–20 s data epochs were obtained from the fetal recording just before delivery, as well as at 6, 8, 10, 15, and 30 min after the start of dynPEEP or SI. No filtering was employed during data analysis, apart from a 48 Hz low-pass filter to remove any 50 Hz electrical interference from signals.

Mean PT micromanometer pressure was matched to the corresponding mean fluid-filled catheter pressure. PT blood flow (i.e. RV output) was derived as the sum of the ductal and total PA flows,^13–15,30,32,36^ with the latter calculated from the product of measured left PA flow and the post mortem total-to-left lung weight ratio.^14,25^ Hemodynamic analyses were performed on ensemble-averaged waveforms typically generated from >12 beats in 5–7 s data epochs, and >25 beats in 10–20 s data epochs.

To obtain the L→R ductal contribution to PA blood flow, all negative segments in the ensemble-averaged ductal waveform were measured, multiplied by the quotient of segment duration and heart period, and then summed to yield total phasic L→R ductal flow. Total phasic R→L ductal flow was then obtained from the difference between net and total L→R ductal flows.^14,25^ As L→R ductal shunting passes entirely to the lungs, the contribution of RV output to PA flow was calculated as the difference between mean PA flow and the magnitude of total L→R ductal shunt flow.^13^ Pulmonary vascular conductance (PVC), the reciprocal of pulmonary vascular resistance, was computed as (total PA flow)/(mean PT pressure – mean LA pressure).

### Statistical analysis

As the impact of dynPEEP on PA blood flow was unknown, a feasibility sample of 7–9/group was chosen based on reduction and a previous preterm lamb study of perinatal changes in PA blood flow following an SI.^12^ Results were analyzed using GraphPad Prism 8 (GraphPad Software Inc., La Jolla, CA). Hemodynamic and blood flow data were analyzed with one-way repeated measures analysis of variance (RM ANOVA) and specific comparisons evaluated by partitioning the within-animal sums of squares into individual degrees of freedom, using a Bonferroni correction as required for multiple comparisons. Data between groups were analyzed using two-way RM ANOVA, with (1) assessment of the interaction between the type of recruitment maneuver and PEEP timepoints, and (2) comparison of differences at PEEP time-points via post hoc Fisher least significant difference tests. Data are expressed as means ± SD and significance was taken at *P* < 0.05.

## Results

### Blood gas and ventilatory variables

After birth, pH, SaO_2_ and PaO_2_ rose, while PaCO_2_ fell, with similar patterns evident in newborn variables between the two groups, except that PaO_2_ tended to be lower after dynPEEP (Table 1). No significant differences were present after birth between the dynPEEP and SI groups in Fio_2_, V_T_, PIP, C_dyn_ or A-aDO_2_, apart from a higher V_T_ at 15 min after dynPEEP (Table 2).

**Table 1:** Perinatal aortic trunk blood variables in the sustained inflation (SI) and dynamic PEEP recruitment (dynPEEP) groups.

Variable	Group	Fetus	NB 10 min	NB 15 min	NB 30 min	*P*_*1*_	*P*_*2*_
**Hb**, g·dl^-1^	SI	12.0 ± 1.4^b^	11.7 ± 0.8	11.6 ± 1.0	11.4 ± 0.9	0.054	0.608
	dynPEEP	12.3 ± 0.8^a^	12.2 ± 0.8	11.8 ± 0.8	11.7 ± 0.8	0.009	
**S**_**a**_**O**_**2**_, %	SI	62.4 ± 9.7^c^	94.7 ± 7.3	95.8 ± 7.2	96.6 ± 4.9	<0.001	0.621
	dynPEEP	59.1 ± 11.8^c^	95.4 ± 4.2	95.5 ± 2.6	94.4 ± 2.6	<0.001	
**pH**	SI	7.34 ± 0.05^c^	7.41 ± 0.05	7.38 ± 0.04	7.33 ± 0.05	<0.001	0.161
	dynPEEP	7.31 ± 0.03^c^	7.37 ± 0.05	7.37 ± 0.03	7.33 ± 0.03	0.002	
**P**_**a**_**O**_**2**_, mmHg	SI	23.7 ± 4.4^c^	81.0 ± 51.9	68.4 ± 27.2	61.6 ± 17.5	0.007	0.065
	dynPEEP	21.9 ± 3.6^c^	53.5 ± 27.9	46.3 ± 16.8	43.3 ± 12.5	0.003	
**P**_**a**_**CO**_**2**_, mmHg	SI	47.6 ± 6.4^c^	34.1 ± 5.2	37.5 ± 4.0	42.6 ± 4.4	<0.001	0.186
	dynPEEP	50.9 ± 4.4^c^	38.1 ± 5.6	37.9 ± 4.1	43.3 ± 3.5	<0.001	

Values are means ± SD; *n* = 9 for dynPEEP and *n* = 7 for SI groups. *P*_*1*_, one-way repeated measures ANOVA, ^a^*P* = 0.009, ^b^*P* = 0.005, ^c^*P* < 0.001, fetal vs. newborn time-points; *P*_*2*_, newborn SI vs. dynPEEP treatment effect, 2-way repeated measures ANOVA.

*Hb* hemoglobin concentration, *S*_*a*_O_*2*_ hemoglobin oxygen saturation, *P*_*a*_O_*2*_ oxygen tension, *P*_*a*_CO_*2*_ carbon dioxide tension, *NB* newborn, *SI* sustained inflation, *dynPEEP* dynamic PEEP recruitment.

**Table 2:** Ventilatory variables in the sustained inflation (SI) and dynamic PEEP recruitment (dynPEEP) groups after birth.

Variable	Group	NB 10 min	NB 15 min	NB 30 min	*P*_*1*_	*P*_*2*_
**Fio**_**2**_, %	SI	30 ± 1	32 ± 10	36 ± 15	0.351	0.978
	dynPEEP	32 ± 6	31 ± 4	34 ± 7	0.318	
**V**_**T**_, ml·kg^−1^	SI	6.9 ± 0.2	5.9 ± 0.2	5.4 ± 0.3	<0.001	0.025
	dynPEEP	7.0 ± 0.1	6.4 ± 0.5^a^	5.8 ± 0.6	<0.001	
**PIP**, cmH_2_O	SI	37 ± 6	31 ± 6	30 ± 6	<0.001	0.188
	dynPEEP	38 ± 3	35 ± 3	33 ± 4	<0.001	
**C**_**dyn**_, ml·cmH_2_O^−1^·kg^−1^	SI	0.25 ± 0.04	0.26 ± 0.05	0.25 ± 0.05	0.121	0.223
	dynPEEP	0.22 ± 0.04	0.23 ± 0.04	0.22 ± 0.05	0.614	
**A-aDO**_**2**_, mmHg	SI	90 ± 49	116 ± 91	142 ± 118	0.126	0.544
	dynPEEP	128 ± 40	128 ± 40	144 ± 56	0.217	

Values are means ± SD; *n* = 9 for dynPEEP and *n* = 7 for SI groups. *P*_*1*_, one-way repeated measures ANOVA; *P*_*2*_, SI vs. dynPEEP treatment effect, 2-way repeated measures ANOVA, ^a^*P* = 0.015, SI vs. dynPEEP.

*Fio*_2_ inspired fractional O_2_ concentration, *V*_T_ tidal volume, *PIP* peak inspiratory pressure, *C*_dyn_ dynamic lung compliance, *A-aDO*_2_ alveolar-arterial O_2_ difference, *NB*, newborn, *SI* sustained inflation, *dynPEEP* dynamic PEEP recruitment.

### Blood pressures and heart rate

**Fig. 1:**
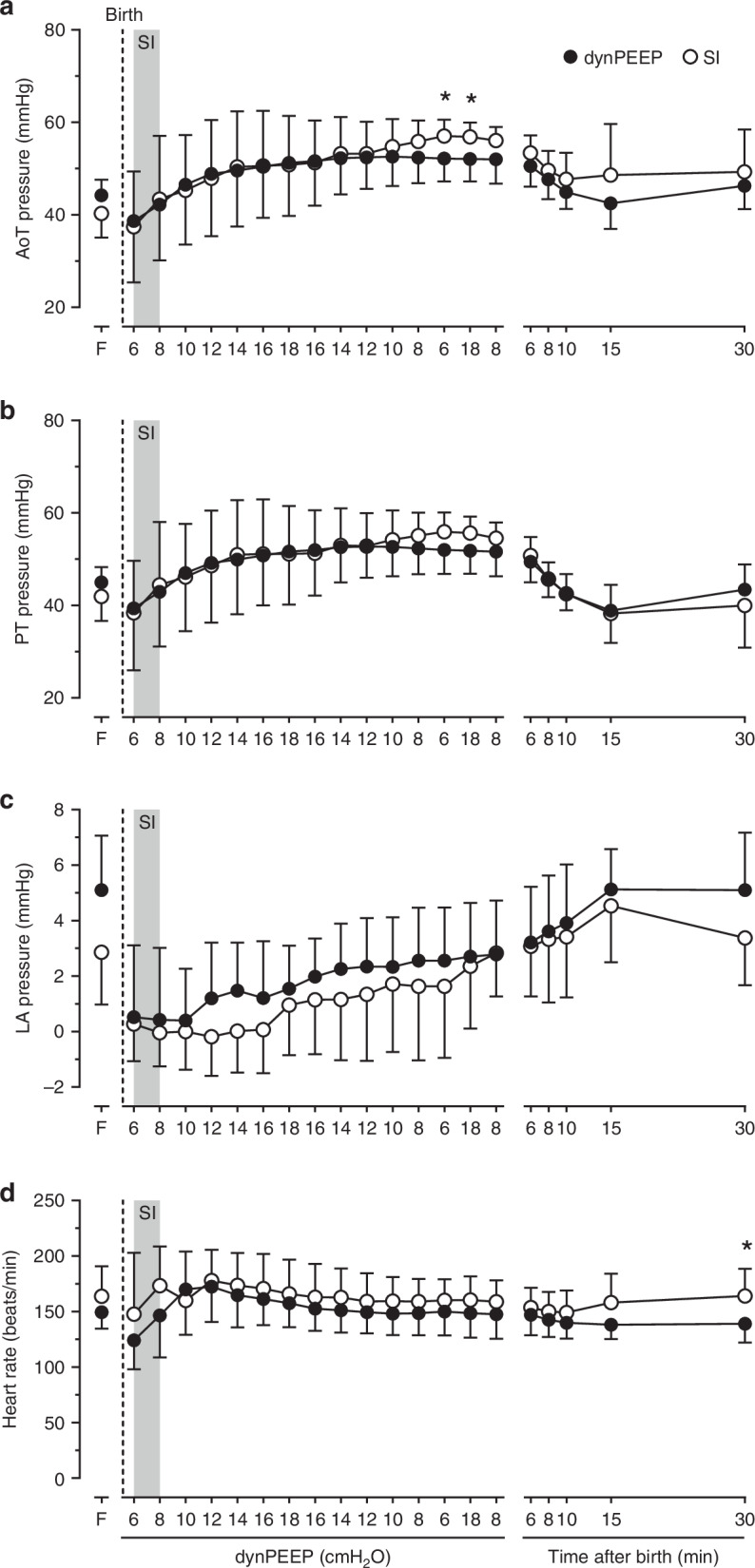
Blood pressures and heart rate with lung recruitment in the birth transition. Changes in mean aortic trunk (AoT, **a**), pulmonary trunk (PT, **b**) and left atrial (LA, **c**) blood pressures and heart rate (**d**) (1) in the baseline fetal state (F), (2) during step changes of PEEP in the dynamic PEEP (dynPEEP) maneuver over ∼4.5 min, or the equivalent time-period after an initial ∼40 s sustained inflation (SI, gray column) followed by mechanical ventilation at a PEEP of 8 cmH_2_O, and (3) in both groups from 6 to 30 min after birth. Values are means ± SD; *n* = 7 for SI and *n* = 9 for dynPEEP groups; * *P* ≤ 0.043, dynPEEP vs. SI (two-way repeated measures ANOVA with post hoc Fisher least significant difference tests). Note that only one limb of the bidirectional SD is displayed to aid visualization.

AoT and PT blood pressures rose during dynPEEP (*P* ≤ 0.007) and the corresponding timepoints in the SI group (*P* ≤ 0.026), before declining between 6 and 10 min in both groups (*P* ≤ 0.031). Arterial pressures did not differ statistically overall between groups (*P* ≥ 0.212), except that AoT pressure was lower after dynPEEP at the end of PEEP de-escalation (*P* = 0.038) and during lung re-recruitment (*P* = 0.032), with similar trends evident in PT pressure (0.096 ≤ *P* ≤ 0.114, Fig. 1a, b). LA pressure increased progressively after birth (*P* ≤ 0.011) and peaked at 15 min, with no difference between groups either during (*P* = 0.350) or after lung recruitment (*P* = 0.476, Fig. 1c). Heart rate rose within the initial minute after birth in both groups (*P* ≤ 0.020) and then remained relatively stable, but was lower in the dynPEEP group at 30 min (*P* = 0.043, Fig. 1d).

### Pulmonary perfusion and vascular conductance

**Fig. 2:**
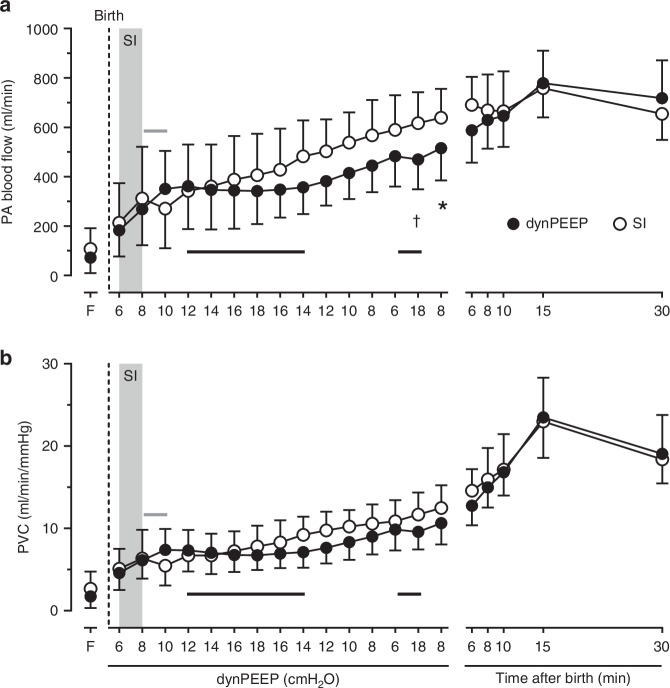
Pulmonary perfusion with lung recruitment in the birth transition. Changes in pulmonary arterial (PA) blood flow (**a**) and pulmonary vascular conductance (PVC, **b**) in the baseline fetal state (F), at varying levels of PEEP during the dynamic PEEP (dynPEEP) maneuver over ∼4.5 min, or the equivalent time-period after an initial ∼40 s sustained inflation (SI, gray column) followed by mechanical ventilation at a PEEP of 8 cmH_2_O, with data depicted in the same format as in Fig. 1. Values are means ± SD; *n* = 7 for SI and *n* = 9 for dynPEEP groups; * *P* = 0.048, ^†^ *P* = 0.021, dynPEEP vs. SI (two-way repeated measures ANOVA with post hoc Fisher least significant difference tests). Note that (1) only one limb of the bidirectional SD is to aid visualization, (2) black horizontal lines are plateaus during dynPEEP and (3) gray horizontal line is plateau after SI.

With dynPEEP, PA blood flow increased linearly between 6 and 10 cmH_2_O during PEEP escalation (*P* < 0.001) and 12–6 cmH_2_O during PEEP de-escalation (*P* = 0.002). However, PA flow displayed a plateau which (1) continued between 12 cmH_2_O PEEP on the escalation limb and 14 cmH_2_O PEEP on the de-escalation limb (*P* = 0.579), and (2) re-emerged with lung re-recruitment (*P* = 0.689). By contrast, PA flow increased during SI (*P* = 0.018), and was then briefly unchanged (*P* = 0.266) before rising linearly in the remaining dynPEEP-equivalent period (*P* < 0.001). PA flow displayed a significant maneuver-PEEP timepoint interaction (*P* < 0.001), with dynPEEP flow lower during (*P* = 0.021) and immediately after lung re-recruitment (*P* = 0.048). Subsequently, PA flow rose further to peak at 15 min after birth (*P* < 0.001), with no difference between groups (*P* = 0.734, Fig. 2a). The patterns of change in PVC after birth resembled those of PA blood flow, including a significant maneuver-PEEP timepoint interaction (*P* < 0.001), manifest as a divergence between the SI and dynPEEP groups during the period of the dynPEEP maneuver (Fig. 2b).

### Right ventricular output

**Fig. 3:**
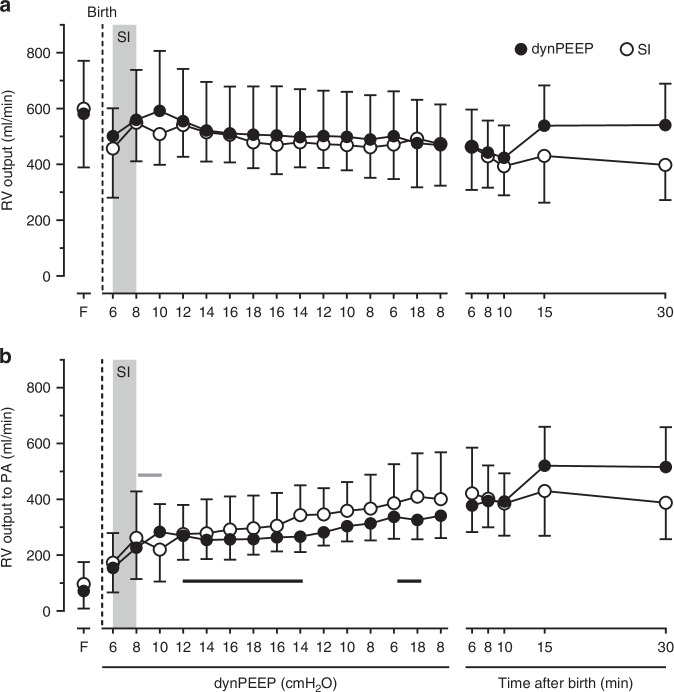
Right ventricular output and its lung distribution with lung recruitment in the birth transition. Changes in right ventricular (RV) output (**a**) and the contribution of RV output to pulmonary arterial (PA) blood flow (**b**) in the baseline fetal state (F), at varying levels of PEEP during the dynamic PEEP (dynPEEP) maneuver over ∼4.5 min, or the equivalent time-period after an initial ∼40 s sustained inflation (SI, gray column) followed by mechanical ventilation at a PEEP of 8 cmH_2_O, with data depicted in the same format as in Fig. 1. Values are means ± SD; *n* = 7 for SI and *n* = 9 for dynPEEP groups. Note that (1) only one limb of the bidirectional SD is to aid visualization, (2) black horizontal lines are plateaus during dynPEEP, and (3) gray horizontal line is plateau after SI.

RV output transiently rose at the start of dynPEEP (*P* = 0.009) and during SI (*P* = 0.034), and then plateaued, with no difference overall between groups during dynPEEP timepoints (*P* = 0.733) or from 6 to 30 min (*P* = 0.248, Fig. 3a). The contribution of RV output to PA flow during dynPEEP increased linearly to a PEEP of 10 cmH_2_O during PEEP escalation (*P* < 0.001) and then displayed a plateau to a PEEP of 14 cmH_2_O on the de-escalation limb (*P* = 0.767), before increasing linearly to a PEEP of 6 cmH_2_O (*P* = 0.005) and plateauing again during lung re-recruitment (*P* = 0.693). By contrast, the RV output component of PA flow increased during SI (*P* = 0.041), and was then briefly unchanged (*P* = 0.330) before rising linearly in the remaining dynPEEP-equivalent period (*P* = 0.003). Subsequently, this contribution rose to a peak at 15 min in the dynPEEP group (*P* = 0.007), but was unchanged from 6 to 30 min in the SI group (*P* = 0.783, Fig. 3b).

### Ductus arteriosus shunting

**Fig. 4:**
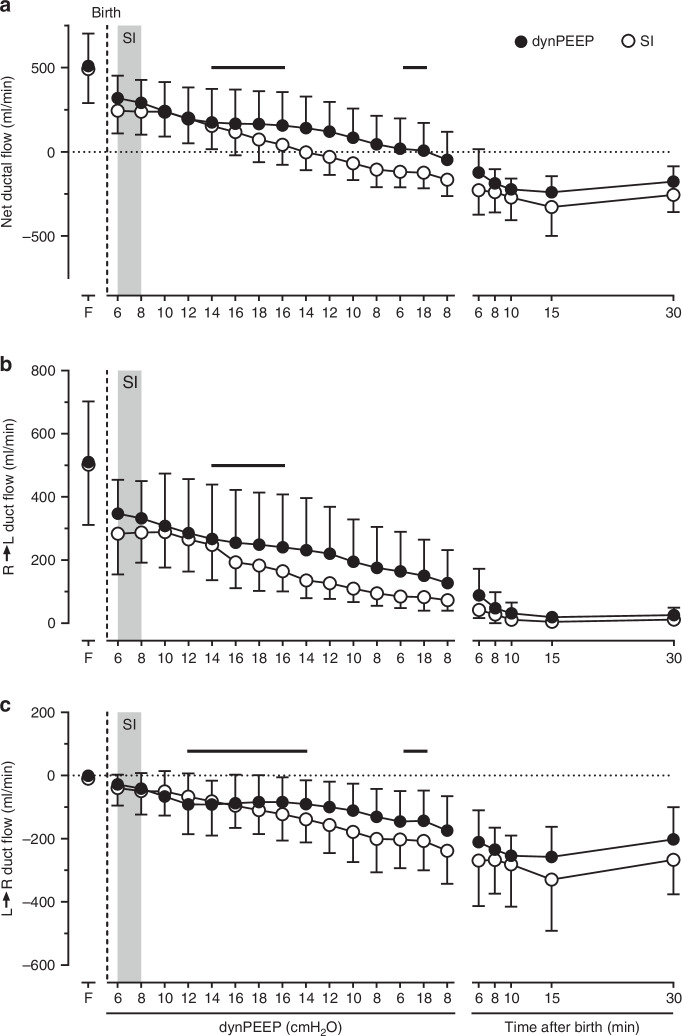
Ductal shunting patterns with lung recruitment in the birth transition. Changes in net (**a**), right-to-left (R→L, **b**) and left-to-right (L→R, **c**) ductal blood flow in the baseline fetal state (F), at varying levels of PEEP during the dynamic PEEP (dynPEEP) maneuver over ∼4.5 min, or the equivalent time-period after an initial ∼40 s sustained inflation (SI, gray column) followed by mechanical ventilation at a PEEP of 8 cmH_2_O, with data depicted in the same format as in Fig. 1. Values are means ± SD; *n* = 7 for SI and *n* = 9 for dynPEEP groups. Note that (1) only one limb of the bidirectional SD is to aid visualization, and (2) black horizontal lines are plateaus during dynPEEP.

During dynPEEP, net (Fig. 4a) and R→L (Fig. 4b) ductal flows decreased linearly as PEEP was increased from 6 to 12 cmH_2_O (*P* ≤ 0.005), then plateaued between 14 cmH_2_O PEEP on the escalation limb and a PEEP of 16 cmH_2_O on the de-escalation limb (*P* ≥ 0.319). These variables declined linearly again as PEEP was reduced to 6 cmH_2_O (*P* < 0.001), but net ductal flow then transiently plateaued with lung re-recruitment (*P* = 0.729). A similar pattern was evident in a progressively greater L→R ductal flow after birth (Fig. 4c). By contrast, these flows were unchanged during the period of SI and the first subsequent epoch (*P* ≥ 0.739), with net and R→L ductal flows then falling, and L→R ductal flow rising, linearly (all *P* < 0.001). Moreover, both net and L→R ductal flow displayed a significant maneuver-PEEP timepoint interaction (*P* ≤ 0.001). However, between 6–30 min after birth, net ductal flow fell to a nadir (*P* = 0.015) and L→R ductal flow peaked (*P* = 0.016), while R→L ductal flow decreased to near-zero (*P* = 0.002), with no difference in responses between groups (*P* ≥ 0.435, Fig. 4a–c).

## Discussion

This study, which has compared the effect of lung recruitment via dynPEEP or a SI on changes in PA and associated central blood flows in preterm lambs undergoing mechanical ventilation during the birth transition, has produced four main findings. First, although dynPEEP and SI were both accompanied by a progressive rise in PA blood flow after birth, PA flow plateaued during dynPEEP at elevated levels of PEEP in the main phase of lung recruitment, as well as during lung re-recruitment at the end of the maneuver, whereas a flattening in PA flow with an SI was only present for a brief period after the end of the inflation. Second, the PA flow pattern with dynPEEP was mirrored in the two sources of this flow, namely RV systolic outflow distributed to the lungs and the mainly diastolic left-to-right (L→R) component of ductal shunt flow, whereas only RV outflow to the lungs was affected by an SI. Third, high levels of PEEP during dynPEEP did not reduce RV output or increase right-to-left (R→L) ductal shunting. Finally, PA blood flow effects of dynPEEP were transitory, with no post-maneuver differences in this flow evident between the dynPEEP and SI groups to 30 min after birth.

As the lungs are fluid-filled in the fetus and pulmonary blood flow is characteristically low in utero,^37–39^ two pivotal steps in the birth transition comprise (1) a rapid clearance of lung liquid from the airways and alveoli to permit establishment of effective ventilation, and (2) an associated and substantial surge in pulmonary perfusion to support gaseous exchange within alveolar-capillary units.^40,41^ During progression of lung aeration in the birth transition, the clearance of lung liquid from the airways accompanying distal movement of a gas-fluid interface can be facilitated by an elevation in airway pressure produced via interventions such as PEEP,^4–7^ an SI applied before the onset of tidal ventilation,^4,8,12^ or incorporation of escalating and de-escalating PEEP lung recruitment into the initial phase of tidal ventilation.^9–11^

An increase in airway pressure may, however, potentially have both beneficial and adverse effects on pulmonary perfusion in the neonatal period. Thus, in conjunction with enhanced arterial oxygenation, an SI augmented the rise in PA blood flow within the initial 3 min after birth.^12^ On the other hand, an increased airway pressure can also reduce pulmonary blood flow via at least three mechanical effects. The first relates to a higher airway (and therefore intra-alveolar) pressure directly increasing external constraint on the pulmonary microvasculature. With a rise in PEEP, alveolar pressure may become similar to or exceed capillary pressure, leading to compression and reduced perfusion of perialveolar capillaries, and thus a fall in pulmonary blood flow, accompanied by increased pulmonary vascular resistance.^42,43^ Secondly, an elevation in airways pressure distends the lungs, and thereby increases the external constraint exerted by the lungs on the heart.^44–46^ This increased constraint may decrease cardiac dimensions and limit ventricular filling, thereby reducing cardiac output via the Frank-Starling mechanism,^42,46^ and thus the level of RV outflow available to perfuse the lungs. Thirdly, greater lung distension secondary to increased airway pressure may compress large central veins, thus reducing systemic venous return into the thorax, and thereby cardiac output.^47^

In the newborn, adverse circulatory effects of an increase in airway pressure appear to predominate following stabilization of the increased PA blood flow occurring with birth. Thus, beyond 20 min after birth, increasing PEEP from 4 to 10 or 12 cmH_2_O in preterm lambs reduced PA blood flow by ∼40%.^17,18^ Moreover, with increases in PEEP instituted after completion of the birth transition, a reduction in PA blood flow persisted following return of PEEP to the baseline level, implying that the elevated airway pressure caused long-lasting changes in the pulmonary vasculature.^17,18^ More specifically, although lung aeration was not measured, the elevated PEEP may have resulted in lung damage from overdistension, with compression of the pulmonary microvasculature secondary to interstitial fluid accumulation, resulting in a reduction of PA blood flow.^17^

On the other hand, our findings suggest that PA blood flow is not decreased by increases in airway pressure occurring with dynPEEP during a rapid post-birth rise in PA flow that peaks within 10–15 min.^12,14,15,30^ Instead, transitory pauses in this rise of PA flow were evident during the highest levels of PEEP in the dynPEEP maneuver, manifested as a plateau between a PEEP of 12 cmH_2_O during PEEP escalation (∼65 s after birth) and a PEEP of 14 cmH_2_O during PEEP de-escalation (∼160 s after birth), as well as during the final lung re-recruitment step (Fig. 2a). Importantly, corresponding plateaus were also evident in PVC (Fig. 2b), suggesting that PA blood flow effects were related to changes in cross-sectional area of the pulmonary vascular bed, and thus the degree of pulmonary vasodilation and/or the extent of vascular recruitment. A plausible basis for these plateaus was that, with progressively greater aeration of the lungs immediately after birth, the PA flow-reducing effect of compression of perialveolar capillaries by increased PEEP^42,43^ was offset by the PA flow-promoting actions of not only greater distension of ventilated alveoli, but also an increase in the distribution of ventilated alveoli throughout the lungs.^48^ It is noteworthy that corresponding plateaus were also present in the two sources of PA blood flow after birth, namely the component of RV output distributed to the lungs (Fig. 3b) and phasic L→R shunt flow crossing the ductus arteriosus (Fig. 4c), implying that the circulatory impact of elevations in PEEP during dynPEEP was not confined to a local lung effect.

In contrast to studies in newborn preterm lambs^17^ or preterm and near-term goats,^21^ which concluded that elevations in PEEP increased R→L ductal shunting, dynPEEP did not augment phasic R→L ductal shunt flow, although a plateau was present in this shunt flow at elevated levels of PEEP (Fig. 4b). Instead, the SI and dynPEEP groups both displayed a typical pattern seen in preterm lambs where, in conjunction with a greater distribution of RV output towards the lungs, both net and phasic R→L ductal shunting progressively decreased within the initial 15 min after birth.^13,14,49,50^ It is likely that two factors contributed to this divergence of findings. Firstly, in both Polglase et al^17^ and Egan and Hessler,^21^ PEEP was increased more than 20 min after birth i.e. beyond the rapid rise in PA flow occurring in the initial 10–15 min after birth.^12,14,15,30^ Secondly, R→L ductal shunting was assessed indirectly via its time fraction within the cardiac cycle using pulsed Doppler ultrasound^17^ or multi-site calculation of blood oxygen content,^21^ rather than via more accurate direct measurement of absolute flow (Fig. 4b).

Unlike the effect of raising PEEP in human newborns ≥3 h after birth,^19,20^ RV output was not decreased during the dynPEEP maneuver, with the overall pattern in RV output not significantly different between the dynPEEP and SI groups (Fig. 3a), and also similar to prior findings in preterm lambs not subjected to any lung recruitment after early cord clamping.^30^ However, although RV output was unchanged during dynPEEP (Fig. 3a), it is unlikely that systemic venous return was maintained throughout this maneuver, as recent data in preterm lambs suggest that an appreciable degree of L→R foramen ovale shunting, which is incorporated into the RV output, can emerge within the initial 5 min after birth and contribute up to ∼20% of PA flow.^50^ As L→R shunting increases pulmonary blood flow at the cost of reducing systemic perfusion,^51,52^ it is thus likely that RV output in newborn lambs of our study exceeded the level of systemic venous return.

As far as we are aware, a transient pause in the increase of PA flow soon after an SI in the birth transition (Fig. 2a) has not previously been described in the literature. However, although not commented upon, PA blood flow was visually unchanged between the 3 and 4 min timepoints in Fig. 3a of Sobotka et al,^12^ a study in preterm lambs where an SI was applied for at least 1 min at birth. Interestingly, the basis of a plateau in PA flow after an SI appeared to differ from that during dynPEEP in our study. Thus, with an SI, a plateau was primarily due to a lack of increase in the distribution of RV output to the lungs, whereas with dynPEEP, a plateau in PA flow was related to a pause in both the distribution of RV outpuut to the lungs (Fig. 3b) and the degree of L→R ductal shunting (Fig. 4c). The differing timing and duration of the relatively brief SI and more prolonged dynPEEP maneuvers is likely to be the main factor underlying this difference, as L→R ductal shunting is relatively minor in the first minute after birth following immediate cord clamping with a non-asphyxial cord clamp-to-ventilation interval, but becomes more pronounced in the ensuing minutes.^13,14,25^

Although the presence of a significant maneuver-PEEP timepoint interaction was manifested as a progressively greater divergence in PA flow (and PVC) between the dynPEPP and SI groups during the time-period of the dynPEEP maneuver, these variables were not statistically different between groups after the end of this maneuver (Fig. 2). This implied that, unlike the persistence of a reduced PA blood flow following return of PEEP to a baseline level after stabilization of post-birth pulmonary perfusion,^17,18^ the PA flow effects of dynPEEP were transient and not associated with long-lasting changes affecting the pulmonary vasculature.

Although increasing PEEP improves arterial oxygenation,^6,18^ the dynPEEP maneuver did not have a significant impact on arterial blood gas variables after birth (Table 1). However, the earliest sample taken after birth in our study was at the 10 min time-point, so the presence of differences between groups during the recruitment maneuvers cannot be excluded. The dynPEEP maneuver also did not result in any substantial differences in the pattern of changes in LA blood pressure (Fig. 1c) or heart rate (Fig. 1d). However, mean AoT blood pressure was lower at the end of initial lung recruitment and during re-recruitment (Fig. 1a), with similar trends evident in PA pressure (Fig. 1b), which is consistent with the finding that an increase in PEEP decreased mean arterial blood pressures in preterm newborn lambs.^16,18^

Our study had four main limitations. First, the extent of instrumentation required to perform the study necessitated general anesthesia and an experimental preparation without surgical closure of a left thoracotomy, which may limit the clinical translation of our findings. Reassuringly, however, baseline blood gas and hemodynamic data in our preparation were similar to those of unanesthetized, chronically-instrumented pre-term fetal lambs.^15,36,53–55^ Moreover, key features of the birth transition, such as a large and rapid rise in PA flow attaining a peak at 10–15 min after birth, and a rapid switchover from fetal R→L to postnatal L→R ductal shunting, were similar to those previously reported in chronically-instrumental fetal lambs.^12,15^ Second, due to the extent of instrumentation and because a left thoracotomy was not surgically closed, we did not directly measure changes in lung aeration. However, using electrical impedance tomography, we have previously demonstrated that, compared to an SI, dynPEEP improves lung aeration, homogeneity of ventilation and lung mechanics, without evidence of increased lung injury.^9,11,56^ Third, neither LV output nor systemic arterial blood flows were measured, so it was not possible to assess the effects of either dynPEEP or an SI on these variables after birth, as well as the associated (1) specific sources of L→R ductal shunting,^13,14^ or (2) contribution of L→R foramen ovale shunting to RV output and PA blood flow.^50^ Finally, preterm lambs were not exposed to lung protective strategies commonly used in clinical practice, such as antenatal glucocorticoids or exogenous surfactant after birth, so the manner in which these therapies may modulate the PA blood flow effects of dynPEEP will need to be defined in future studies.

The findings of this study have implications for a common clinical conundrum, namely that reversing lung atelectasis with PEEP will improve pulmonary perfusion, but if the increase in PEEP is excessive, then pulmonary perfusion may be impeded. In clinical practice, dynPEEP strategies aim to embed caution by being transitory and step-wise to allow assessment of response. This approach is based on the improved aeration and favorable clinical outcomes seen with use of open-lung recruitment maneuvers to reverse atelectasis during high-frequency oscillatory ventilation in neonates.^57,58^ Whether this effect in the already-aerated lung is translatable to the clinical birth transition is unknown. However, a large current clinical trial of dynPEEP at birth (Clinicaltrials.gov Registry: NCT04372953) may provide information on the efficacy and safety of this approach in preterm infants.

In summary, this study has demonstrated that a dynPEEP maneuver after birth in a preterm ovine model is accompanied by a progressive rise in PA blood flow, with transient flow plateaus at elevated levels of PEEP in the main phase of this maneuver, as well as during lung re-recruitment at the end of the maneuver, without a decrease in RV output or increase in R→L ductal shunting.

## Supplementary information

Supplement 2

## Data Availability

All data, including raw data used for all figures and analyses is available upon request to the corresponding author from six months following article publication to researchers who provide a methodologically sound proposal, with approval by an independent review committee (“learned intermediary”). Proposals should be directed to joe.smolich@mcri.edu.au to gain data access. Data requestors will need to sign a data access or material transfer agreement approved by MCRI.
