# Supplementary material for: Pulmonary perfusion with dynamic PEEP recruitment or sustained inflation at birth in preterm lambs
Source: Pediatr Res. 2025 Jun 13;99(2):773–82. doi: 10.1038/s41390-025-04183-x (PMC12956736; doi:10.1038/s41390-025-04183-x)
Supplement: Supplementary file 1 — Supplement 2 [file 41390_2025_4183_MOESM1_ESM.pdf]

Supplement 2

Surgical preparation and monitoring of ewe

Pregnant ewes were obtained from an MCRI-accredited supplier and housed in the MCRI Translational Research Unit with standard husbandry care. On the morning of the experimental study, ewes were premedicated with i.m. ketamine (5 mg/kg) and xylazine (0.1 mg/kg), and then anesthetized with 4% isoflurane given by mask. After intubation of the trachea with a cuffed endotracheal tube, a surgical plane of anesthesia was maintained with isoflurane (0.5-2%) and nitrous oxide (10-20%) delivered by ventilator in O_2-enriched air, supplemented by infusion of ketamine (1-1.5 mg/kg/hr), midazolam (0.1-0.15 mg/kg/hr) and fentanyl (2-2.5 μg/kg/hr) via a right external jugular venous cannula. Transcutaneous oxygen saturation was monitored continuously with a pulse-oximetry sensor applied to the ear or cheek. The right common carotid artery was cannulated through a midline neck incision for monitoring of blood pressure and regular blood gas analysis (ABL800, Radiometer, Copenhagen, Denmark), with ventilation of the ewe adjusted to maintain arterial O_2 tension (P_ao_2) at 100-120 mmHg and CO_2 tension (P_aco_2) at 35-40 mmHg.
